# Genome-wide insights into the genetic history of human populations

**DOI:** 10.1186/s13323-015-0024-0

**Published:** 2015-04-01

**Authors:** Irina Pugach, Mark Stoneking

**Affiliations:** Department of Evolutionary Genetics, Max Planck Institute for Evolutionary Anthropology, Deutscher Platz 6, D04103 Leipzig, Germany

**Keywords:** Demographic history, Genome-wide data, Ascertainment bias

## Abstract

Although mtDNA and the non-recombining Y chromosome (NRY) studies continue to provide valuable insights into the genetic history of human populations, recent technical, methodological and computational advances and the increasing availability of large-scale, genome-wide data from contemporary human populations around the world promise to reveal new aspects, resolve finer points, and provide a more detailed look at our past demographic history. Genome-wide data are particularly useful for inferring migrations, admixture, and fine structure, as well as for estimating population divergence and admixture times and fluctuations in effective population sizes. In this review, we highlight some of the stories that have emerged from the analyses of genome-wide SNP genotyping data concerning the human history of Southern Africa, India, Oceania, Island South East Asia, Europe and the Americas and comment on possible future study directions. We also discuss advantages and drawbacks of using SNP-arrays, with a particular focus on the ascertainment bias, and ways to circumvent it.

## Review

### Introduction

Studies of the genetic history of human populations have relied largely on variation in the single-locus, uniparentally inherited mtDNA and non-recombining Y chromosome (NRY). While mtDNA and the NRY continue to provide valuable insights (as reviewed elsewhere in this issue), especially with the advent of new sequencing methods based on next-generation platforms, genome-wide data are increasingly supplementing and supplanting single-locus studies. Genome-wide data generally provide more reliable insights into population history in that they are based on analyses of many independent loci, whereas the history of a single locus may depart from that of the population as a whole because of chance events or selection influencing that locus. Genome-wide data are particularly useful for inferring population divergence times, migration and admixture (especially the timing of such events), changes in population size, and other aspects of demographic history. In this review, we focus on some of the stories, that is, aspects of human population history as revealed by analyses of genome-wide data from contemporary human populations that we find of particular interest, rather than providing a comprehensive overview of methods and results. There are certainly other interesting studies which we do not discuss in this review [1-9]; other additional references are provided where relevant. We also do not consider the impact of selection or insights from analyses of ancient DNA; although these are certainly relevant, they are covered elsewhere in this issue. Genome-wide analyses began with studies of short-tandem repeat (STR) loci (also known as microsatellites), and while these provided some important insights into human population history [10-13], STR studies have been largely replaced by SNP data obtained from microarrays, as well as increasingly by genomic sequencing. We begin with a few general comments and then provide some examples of the types of insights that have resulted from genome-wide studies.

**Figure 1:**
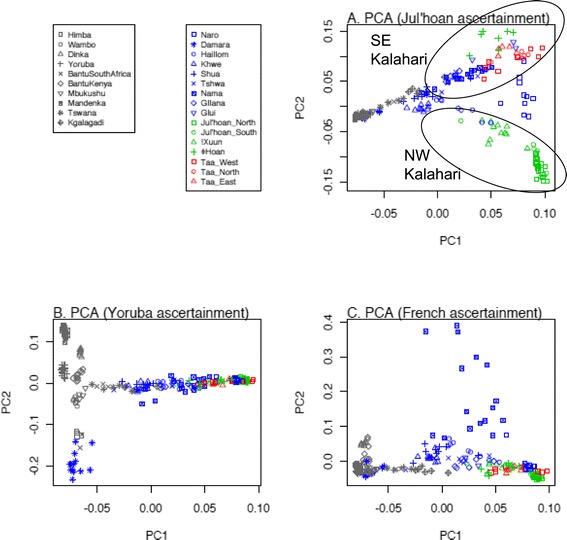
**PC plots for Southern African Khoisan**-**speaking and Bantu**-**speaking individuals genotyped on the Human Origins Array. (A)** Results based on SNPs ascertained in a Khoisan individual. Ellipses indicate groups from either the NW or SE Kalahari Basin. **(B)** Results based on SNPs ascertained in a Yoruba individual. **(C)** Results based on SNPs ascertained in a French individual. Reprinted with permission from Pickrell *et al*. [25]. PCA, principal component analysis; SE, southeastern, NW, northwestern.

Whole genome sequencing is, at the time, we write this, still too costly (in terms of time and money) to be applied to large numbers of individuals from large numbers of populations - although the situation is rapidly changing. For now, most genome-wide data comes from the so-called ‘SNP chips’, which are microarrays containing probes to hundreds of thousands (or even millions) of SNPs. DNA samples can be genotyped quickly and reliably at relatively low cost; however, SNP chips are not without their drawbacks, the main one being ascertainment bias. Ascertainment bias refers to how the SNPs were chosen for inclusion on the chips and inevitably arises because, by definition, only sites known to be polymorphic in at least one population are interrogated by the microarray. And since European populations (or those of recent European origin, such as European-Americans) are the most studied, most SNPs on the commercial SNP chips were ascertained to be polymorphic in Europeans. This has several important consequences. First, heterozygosity in European populations will be over-estimated relative to non-European populations (see, for example, Figure 1 in López Herráez *et al*.) [14]. Second, the allele frequency distribution based on SNP chip data will be skewed towards alleles of intermediate frequency. This means that approaches for inferring aspects of demographic history based on the allele frequency distribution or related properties such as the average heterozygosity of a population (for example, [15-21]) cannot be used with SNP chip data. Third, different SNP chips ascertain SNPs for different purposes; for example, some use ‘tag’ SNPs that are spaced evenly across the genome, which means that such data have limited power for making inferences based on linkage disequilibrium (non-random associations between genotypes at different SNPs, which can provide a lot of information for certain demographic inferences). Still, there are ways to work around the ascertainment bias problem. One approach is to incorporate the ascertainment bias into the demographic inference procedure, and examples will be discussed later [22,23]. This sort of approach works best when the method of SNP ascertainment is either known or can be estimated from the data, but such information is not always readily available or provided by companies. A welcome counterexample is the Affymetrix Human Origins Array [24], which contains 11 different sets of SNPs, each ascertained on the basis of being heterozygous in a single genome sequence from each of the 11 different populations. Analyzing the data from the different SNP panels separately can lead to interesting insights, and an example will be discussed below concerning Southern African populations [25]. Nonetheless, it is important to keep in mind that with SNP chip data, you only find out about the polymorphisms you already know about; complete genome sequence data shows you what you have thereby missed, which can be considerable [26].

### Southern African Khoisan-speaking groups

MtDNA and Y-chromosome analyses have shown that Khoisan-speaking groups (that is, those speaking non-Bantu languages that use click consonants) from Southern Africa harbor some of the deepest rooting lineages among extant human populations [27,28], and genome-wide data confirm this picture [29,30]. However, there is more to the story than the earliest divergence among human populations. Khoisan-speaking groups harbor extensive linguistic, cultural, and phenotypic diversity: Khoisan languages are currently classified into three families that have no demonstrable relationship with one another [31]; Khoisan-speaking groups include not only foragers but also food producers (both pastoralist and agricultural groups); and while some Khoisan-speaking groups conform to the stereotypical phenotype of having on average small stature, light skin pigmentation, and so on, others are on average taller and have darker skin pigmentation and more closely resemble Bantu-speaking groups [32]. The extensive linguistic, cultural, and phenotypic diversity of Khoisan-speaking groups is also mirrored in their genetic diversity. Genome sequences from two Khoisan-speaking individuals exhibit more nucleotide differences between them than do a genome sequence from a European compared to an Asian [30], and two studies of genome-wide SNP data [25,33] have found deep genetic structure among Khoisan-speaking groups that is estimated to reflect a separation of approximately 30,000 years. Interestingly, this structure does not reflect linguistic differences among groups but rather seems to correspond roughly to a geographical separation of northwestern from southeastern Kalahari groups (Figure 1A).

As the data depicted in Figure 1A were obtained with the Human Origins Array, which consists of different SNP panels with different ascertainment, the effects of different ascertainment on the results were examined [25]. The data in Figure 1A are for SNPs ascertained on the basis of heterozygosity in a single genome sequence from a Ju|'hoan individual; note that PC1 reflects largely a separation between Bantu-speaking and Khoisan-speaking groups, while PC2 reflects genetic differences among Khoisan-speaking groups. If one instead analyzes SNPs ascertained from a Yoruba (Figure 1B) or French (Figure 1C) individual, while PC1 remains largely the same, PC2 is quite different. With SNPs ascertained from a Yoruba individual (Figure 1B), the Khoisan-speaking groups now exhibit little in the way of genetic differences in PC2; instead, PC2 distinguishes Bantu-speaking groups from one another (along with the Damara, who genetically are more similar to Bantu-speaking groups than to other Khoisan-speaking groups [25]). And with SNPs ascertained from a French individual (Figure 1C), PC2 distinguishes the Nama from other groups, which probably reflects more Eurasian ancestry in the Nama than in the other groups. Thus, how SNPs were ascertained has a profound influence on the results of the principal component (PC) analysis. Still, ascertainment bias should not always be viewed as problematic; as long as one is aware of the ascertainment bias, one can actually utilize it to learn more about the genetic relationships and structure of the populations analyzed, as exemplified in Figure 1A,B,C.

A subsequent re-analysis of the data in this study [34] was carried out using new methods based on linkage disequilibrium (LD) to infer and date admixture events [35]. The basic idea is that an admixture event between two populations will introduce LD that will then break down over time due to recombination and new mutations, and there are a variety of methods for detecting and dating admixture events based on the breakdown of LD [35-37]. The results surprisingly showed that all Khoisan-speaking groups harbor a signature of Western Eurasian ancestry (most closely related to European and Middle Eastern groups) that dates to about 900 to 1,800 years ago, well before recent European colonization of the African continent [34]. Further investigation showed that a related signature of Western Eurasian ancestry also occurs in Eastern African populations; the Western Eurasian ancestry in Eastern Africa is both older than that in Southern Africa (dating to approximately 3,000 years ago) and is a better proxy for the Western Eurasian ancestry in Southern Africa than is provided by contemporary Western Eurasian groups. These results suggest a scenario in which there was a migration from Western Eurasia to Eastern Africa followed by admixture about 3,000 years ago, and then, a subsequent migration from Eastern Africa to Southern Africa followed by admixture around 900 to 1,800 years ago, which contributed both Eastern African and Western Eurasian ancestry to Southern African groups.

A reasonable test of this hypothesis would be to determine if the amount of Eastern African ancestry is correlated with the amount of Western Eurasian ancestry in Southern African groups. Unfortunately, it was not possible to carry out this test, because with the SNP chip data, Eastern African ancestry cannot be reliably distinguished from Western African ancestry. This is because the detection of ancestry from a specific population relies on the existence of sufficient genetic drift since the divergence of that population from other populations to create different allele frequencies, and thus a distinct genetic signature for that ancestry. Eastern and Western African populations have not experienced sufficient drift since their divergence to create distinctive genetic signatures of their ancestry, whereas the bottleneck associated with the migration of modern humans out of Africa has created a distinctive genetic signature for non-African populations, making it very easy to detect Western Eurasian ancestry in African populations. All of the Khoisan-speaking groups studied carry recent Western African ancestry from Bantu-speaking groups (as evidenced by mtDNA and Y-chromosome studies [27,38-40] that arrived in Southern Africa in the past 2,000 years, so any ‘non-Khoisan’ African ancestry in the genome-wide data could be of Western African origin, Eastern African origin, or both. This inability to distinguish between Eastern and Western African ancestry is presumably a limitation of the lower resolution of the SNP chip data; when sufficient whole genome sequences become available, it will probably then be possible to distinguish Eastern from Western African ancestry and hence revisit this issue. In the meantime, other genetic data, such as a Y-chromosome marker [41] and a lactase persistence variant [42,43], do support the hypothesis of a migration from Eastern Africa to Southern Africa that probably brought pastoralism to Southern Africa. Thus, contrary to the stereotypical view of Khoisan-speaking groups having existed for a long time in isolation from other groups, there have been (at least) two prehistoric migrations that have had a genetic impact on these groups: a migration of pastoralists from Eastern Africa and the migration of Bantu-speaking groups. In addition, we refer the reader to other relevant genome-wide studies of demographic history of African populations and populations currently residing at the ‘out of Africa’ crossroads [44-50], that we do not discuss in detail here.

### Genetic prehistory of India

India harbors extensive linguistic and cultural diversity, and genome-wide studies have helped shed light on the origins of some of this diversity. In particular, the linguistic and cultural data indicate contributions from outside India; were these accompanied by genetic contributions as well? For example, Indo-European (IE) languages are predominant in northern India and are related to languages elsewhere in Eurasia, while Dravidian languages are predominant in southern India and are restricted to South Asia. Also, agriculture seems to have spread into India from elsewhere in western Asia, possibly concomitantly with IE languages [51]. Was the spread of these and other cultural traits accompanied by an actual migration of people, who also contributed genetic ancestry to current Indian populations, or did languages and farming spread via cultural diffusion?

A study of genome-wide SNP data in 25 groups from across India found strong support for two distinct sources of genetic ancestry [52]. The first, dubbed ‘Ancestral North Indian’ (ANI) because it is predominant in northern India, shows affinities with contemporary populations from Europe, the Middle East, and Central Asia. The second, dubbed ‘Ancestral South Indian’ (ASI) because it is predominant in southern India, does not show such affinities; indeed, ASI, ANI, and East Asian genetic ancestry are all equally distinct from one another. Across India, from North to South, there is a gradient of decreasing ANI and increasing ASI ancestry. These results suggest that ASI represents an older, indigenous Indian ancestry, and that ANI represents a later migration of people into northern India from elsewhere. While it is tempting to associate the spread of ANI ancestry with the spread of IE languages and/or farming, it must be kept in mind that the admixture signal between ANI and ASI ancestry was not dated, so the ANI ancestry could instead be associated with older or more recent migrations.

**Figure 2:**
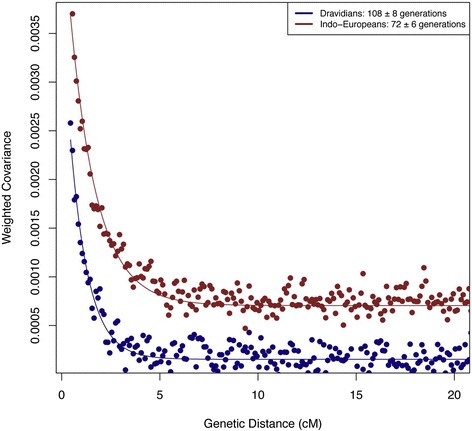
**Analysis of admixture LD in Indo**-**European speakers and Dravidian speakers from India.** The plot shows the weighted covariance (with weights corresponding to relative allele frequencies in the ANI and ASI components), calculated for each pair of SNPs and used as a measure of LD *vs*. genetic distance between these SNPs; the fitted line is used to obtain the time estimates (in generations) since the admixture event. Reprinted with permission from Moorjani *et al*. [53].

A later follow-up study of the same data did date the onset of ANI-ASI admixture via an analysis of patterns of admixture LD [53]. Briefly, the methods used [35] involve plotting the weighted covariance (where the weights reflect the allele frequency differences in the parental groups involved in the admixture) between pairs of SNPs *vs*. how far apart they are on the same chromosome and fitting an exponential equation that can then be used to estimate the number of generations that have elapsed since admixture (Figure 2). The results indicate that the admixture occurred at various times between about 2,000 and 4,000 years ago and generally earlier in IE-speaking groups than in Dravidian-speaking groups. An important caveat to note is that this sort of analysis assumes a single pulse of admixture, so if admixture has been continuous over time or has occurred multiple times, the resulting dates are only for the most recent admixture. So, the actual migration that brought ANI ancestry to India could have occurred considerably earlier than 2,000 to 4,000 years ago. Moreover, a single pulse of admixture does not provide a good fit to the results for some of the populations, suggesting multiple waves of migration. For example, the more recent signal of admixture in northern IE-speaking groups than in southern Dravidian-speaking groups is not consistent with a single wave of migration spreading from north to south, as then one would expect older admixture dates in the north and more recent admixture dates in the south. It seems likely that there has been additional gene flow into northern India from ANI-related populations that was more recent than the first migration to bring ANI ancestry to India. It is to be anticipated that full genome sequence data will shed further light, although the first such large-scale study in India [54] focused on disease-related aspects rather than these questions about demographic history. Nonetheless, the overall time frame suggested by the analyses of the genome-wide SNP data is consistent with the hypothesis that ANI ancestry was brought to India along with IE languages and farming. It does seem rather reasonable to assume that when people migrate, they bring with them their language and cultural practices such as farming [55]; another such example is the Austronesian expansion, discussed below.

### Origins of the Romani

The Romani (also known as Roma and sometimes called ‘Gypsies’ by outsiders) are the largest ethnic minority in Europe, numbering an estimated 10 to 12 million people. There are a wide variety of Romani dialects, religions, and social practices, but the Romani are united by a shared history of having migrated from India around 1,000 to 1,500 years ago. Linguistics, cultural practices, and limited genetic studies support this view of an Indian origin of the Romani, but many details (such as the likely geographic source in India, the route of migration, and the amount of admixture with other populations along the way from India to Europe) remain unknown. Two studies of genome-wide SNP data have recently provided additional insights into the origins of the Romani [22,56]. These studies used different datasets and somewhat different methods: one analyzed admixture LD [56] as described above; while the other used approximate Bayesian computation (ABC) to make detailed inferences about Romani demographic history [22]. ABC is a simulation-based approach that can be used to both infer which of several competing models is the best explanation for the data, as well as then estimate demographic parameters of interest (such as population divergence times, population size changes, and migration events). To choose among different models of the branching structure of population history, genome-wide data are simulated under each model, summary statistics (based on diversity within populations and/or divergence among populations) are calculated from the simulated data, and then, the summary statistics for the simulated data are compared to those for the observed data. This procedure is repeated, typically a few million times or so, and the support for each model is evaluated; the model receiving the highest support (by showing the smallest differences between the simulated and observed data) is taken as the most likely model. For a specific branching history, additional demographic parameters of interest are then estimated by another round of simulations, in which a prior distribution is assumed for each parameter of interest. A value for each parameter is then drawn from the prior distribution, data are simulated with this set of parameter values, and the resulting summary statistics are calculated. This is repeated a few million times, and the sets of parameter values that provide simulated summary statistics that come closest to the observed values for those statistics are retained (typically, the best 0.1% of a few million simulations are retained). The resulting distributions for the parameter values are taken as representing the likely ranges for those parameters.

**Figure 3:**
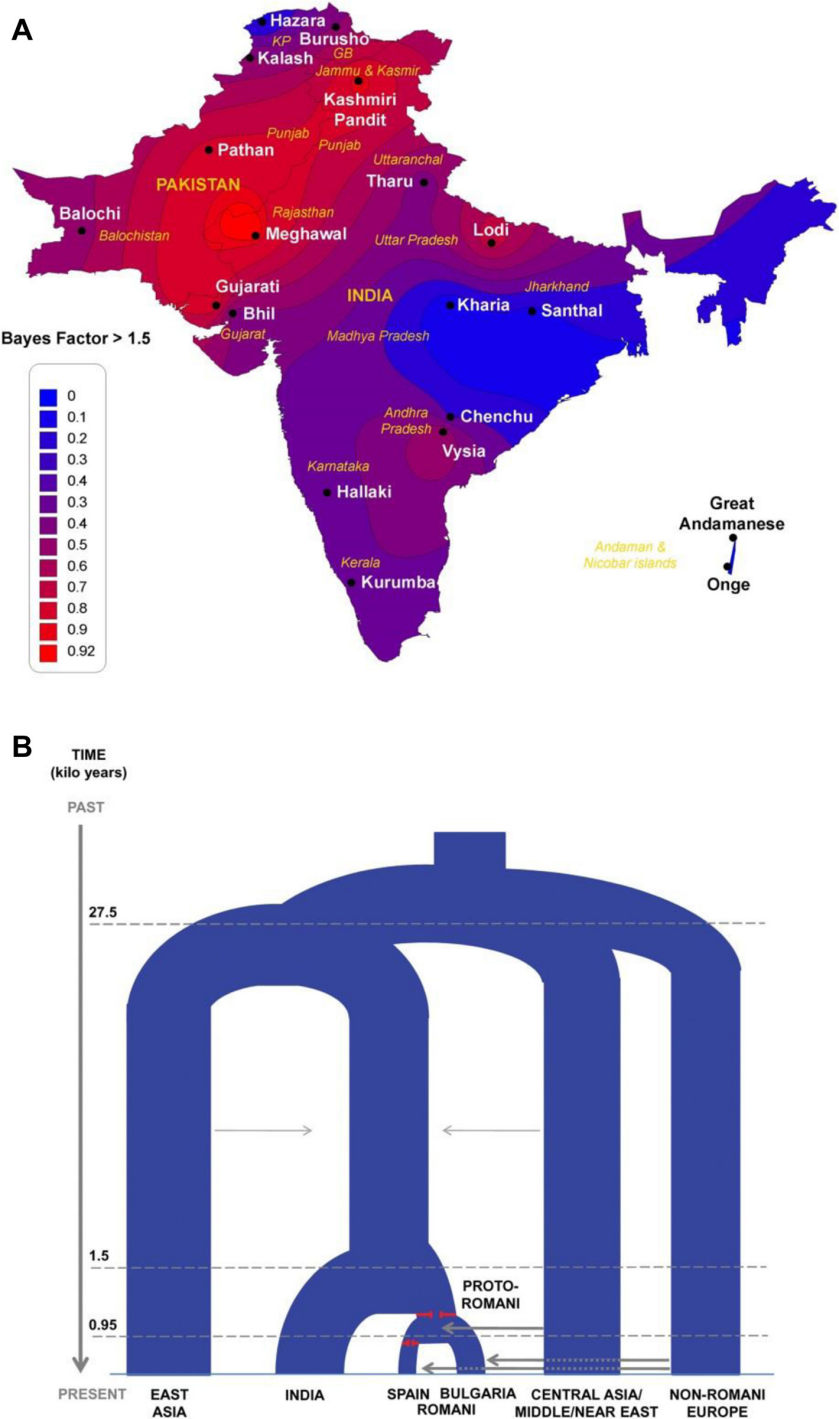
**Origins and history of the Romani, based on demographic inferences from genome**-**wide SNP data. (A)** Heat map showing the percentage of times in the ABC analysis that a particular region was inferred as the most likely source of the Romani. **(B)** Inferred demographic history of the Romani, based on ABC analyses. Branch widths are proportional to effective population sizes; red lines indicate bottleneck events, and arrows indicate migration events. Reprinted with permission from Mendizabal *et al*. [22].

When applied to genome-wide data for the Romani and reference populations from Western Eurasia and South Asia, both the admixture LD and the ABC approaches come to broadly similar conclusions. The Romani likely originated from somewhere in northwestern India (Figure 3), even though populations were not actually sampled from the region inferred to be the source, some 1,500 years ago. There was an associated strong reduction in population size (bottleneck), followed by migration from India westward. There was some admixture with Central Asia and Middle Eastern populations but much more admixture in the Balkans about 900 years ago (Figure 3). This was followed by a major increase in population size associated with the spread of the ancestors of the Romani across Europe, and then (as might be expected), the history becomes much more complicated, with highly variable patterns of admixture between Romani and non-Romani in different parts of Europe and evidence of further bottlenecks, continuing to the present. The genome-wide data thus further extend and refine the historical record of the Romani and help illuminate their rich and complex history.

### Oceania

Oceania holds a unique place in the human history of the world, as the genetic diversity in this region has been shaped by at least two major human migrations - the first out-of-Africa migration and the last pre-European dispersal of people, known as the Austronesian expansion. Australia and New Guinea, which up until 8,000 years ago, were joined into a single landmass called Sahul and were first settled during the expansion of modern humans out of Africa; the earliest sites documenting the presence of anatomically modern humans are dated to approximately 50,000 years ago in Australia [57] and approximately 40,000 years ago in New Guinea [58]. Details of the initial colonization of Oceania, that is, a single or multiple waves of settlers and the route and timing of the migration(s), were fiercely debated, and studies based mainly on mtDNA and NRY variation often provided conflicting results. Most studies supported different origins for Australians and New Guineans as they found no genetic affinity between them [59-63], while others - including those based on Alu insertion polymorphisms [64,65] and *Helicobacter pylori* [66] - provided evidence for deep common ancestry. It was not until genome-wide data were obtained, which allowed for greater depth and resolution, that these questions were finally answered decisively.

Two recent studies which analyzed dense SNP genotyping data from aboriginal Australians and New Guineans [67,68], although confirming a deep divergence of indigenous Australians from the other world populations, did identify highlanders of Papua New Guinea as their closest relatives. Early settlement of the continent, as attested by archeological dates [57], as well as high genetic differentiation of aboriginal Australians and Papua New Guineans, led some researchers to suggest that the dispersal into Near Oceania was part of a separate earlier out-of-Africa migration than the one that settled other regions of the world. We now know that this theory has little merit, as it was tested along with the two other hypotheses for the origins of New Guineans, using approximately 1 million SNPs from Oceanian populations [23]. Three models were tested, and the demographic model that received the highest support simulated a split of New Guineans from Eurasians (estimated posterior probability of 0.74); the posterior probability of a New Guinea split from East Asians was only 0.24, and a direct split of New Guineans from Africans had virtually no support at all (*P* = 0.02).

Although genome-wide data made it possible to reject an ‘early’ dispersal hypothesis, identifying a possible route of the dispersal remains a challenging task, as any archeological evidence for the southern coastal route out of Africa would have been swallowed by rising sea levels at the end of last glaciation, and the genetic record erased by subsequent migrations. In addition to the Australian aboriginals and the highlanders of New Guinea, the so-called Negrito groups of Malaysia and the Philippines and the Andamanese Islanders are thought to be the only direct descendants of the out-of-Africa diaspora via a southern route, while the other populations who live in Southeast Asia today have been shown to have arrived later by a separate dispersal from the north [69-71]. Genetic links between the aboriginal Australians and the Filipino Negrito groups have been suggested, initially based on NRY data [72], and such evidence has been considerably strengthened with genome-wide data, which revealed a close affinity of aboriginal Australians and Papua New Guineans to the Aeta [71] and the Mamanwa [68,70] Negrito groups from the Philippines. Furthermore, large-scale genotyping data allowed for the first time an estimate of the time of divergence between the aboriginal Australians and the other world populations. Using the correlation in genome-wide LD patterns between populations to estimate their time of divergence [73], Pugach *et al*. estimated that Eurasians and the populations of greater Australia diverged from African populations 66 kya, while the split between Australians and New Guineans from the Eurasians was dated to around 43 kya, and the divergence between the Australians, New Guineans, and the Mamanwa Negrito group was estimated to have occurred 36 kya [68]. This date of 36 kya is in broad agreement with the date of divergence estimated from the bacterium *H. pylori* [66]. Interestingly, this date implies that the aboriginal Australians and the New Guineans split soon after the initial dispersal into Sahul, while it was still one landmass, and not when the rising sea waters separated the island of New Guinea from Australia around 8,000 years ago.

The next chapter in the history of Oceania started tens of thousands years later with a large-scale Austronesian expansion, which began about 4,500 years ago from Taiwan [55,74-77], proceeded through the Philippines to Indonesia and spread as far west as Madagascar and as far east as the furthest islands of Polynesia. The impact of this expansion on Island Southeast Asia will be discussed in the next section, while here, we review key points concerning Near and Remote Oceania.

While the first Paleolithic expansion into Near Oceania brought modern humans to Australia, New Guinea, and the nearby archipelagos (together known as Melanesia), the latter Holocene dispersal was of people who must have been in possession of more advanced seafaring skills and technologies, which enabled them to venture further into Remote Oceania, and colonize islands scattered over the Pacific Ocean and often separated from each other by thousands of kilometers of open water. Earlier mtDNA and NRY studies provided evidence that once they reached Melanesia, Austronesian speakers started mixing with the indigenous Papuan-speaking populations and that this newly admixed population subsequently expanded into Remote Oceania [78-85]. This extensive mixing prior to the expansion of populations of Asian and Papuan ancestry was reflected in the ‘Slow Boat’ model of Polynesian origins [62]. Furthermore, this admixture was shown to be sex-biased, as most mtDNAs in Island Melanesia and Polynesia today are of Asian origin, while the NRYs are predominantly New Guinean [78,83], in keeping with an inferred matrilocal residence pattern for Austronesian communities [86,87]. This paints a fairly uncomplicated picture of a single ancient initial colonization, followed by a single dispersal from Taiwan to Island Melanesia leading to extensive mixing with the indigenous communities prior to expansion into Remote Oceania. However, this simple scenario, while providing a framework for understanding the major genetic legacy of human dispersals into Oceania, does not explain everything, as some archeological, linguistic and genetic evidence suggest a more complex story. For example, the discontinuous distribution of a distinctive style of pottery known as Lapita that is associated with Austronesian expansion into the Pacific, complicated linguistic patterns [74-77], and the presence of some genetic outliers, for example, the island of Santa Cruz in the Remote Oceania, where Papuan mtDNA and Y chromosomes haplogroups are prevalent [88-90], indicate that the simple two-wave scenario is incomplete. For instance, the island of Santa Cruz, one of the first across the border in Remote Oceania, has much higher Papuan genetic ancestry than any other island in Remote Oceania [88-90] and thus does not appear to simply be the first stop of ancient voyagers as they proceeded to colonize Remote Oceania. In-depth studies of regional variation are needed to provide greater details concerning precise routes of colonization, potential additional movements of people, and contact between populations following expansion into Remote Oceania.

**Figure 4:**
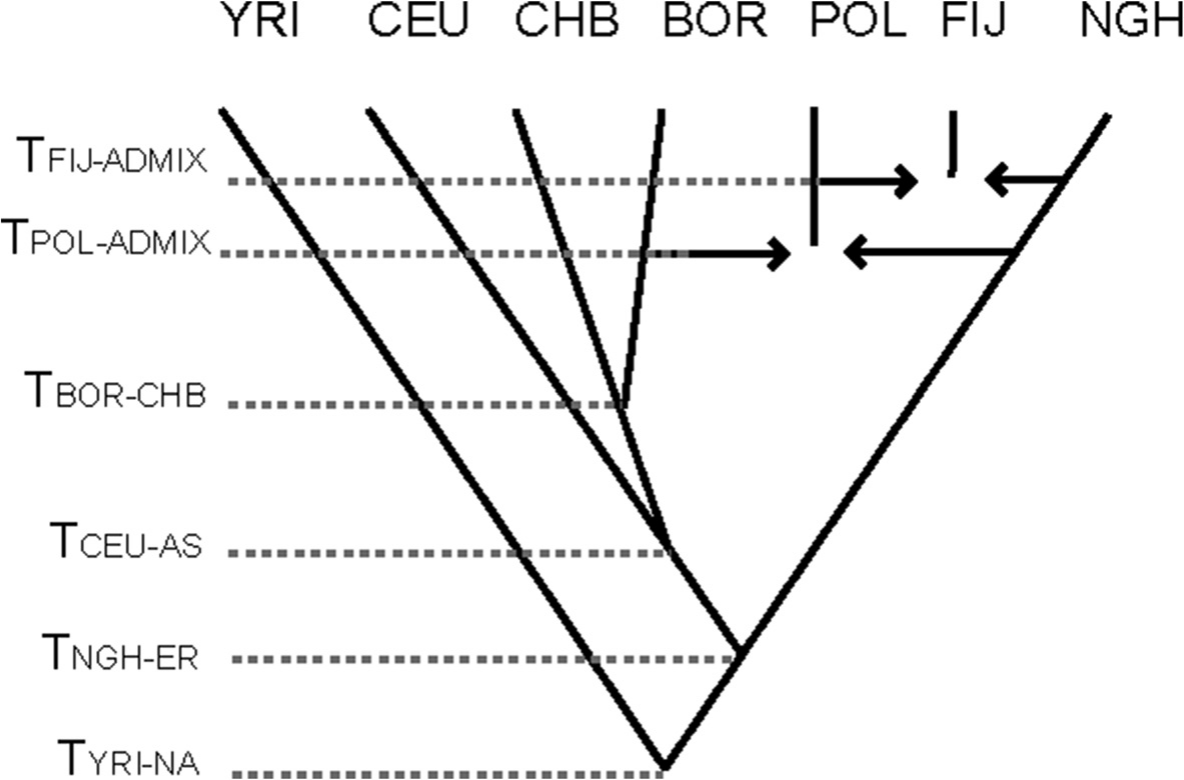
**Preferred model for the human history of Oceania.** Model depicts the most likely tree topology, based on tested competing hypotheses on the initial out-of-Africa split between sub-Saharan Africans (YRI), Europeans (CEU), East Asians (Chinese, CHB), and New Guinea Highlanders (NGH); admixture of Polynesians (POL) and the origins of Fijians (FIJ). NA, non-Africans; AS, Asians; ER, Eurasians. Reprinted with permission from Wollstein *et al*. [23].

Very important insights into the origins of Polynesians were recently made possible by a study of nearly 1 million SNPs genotyped in populations of New Guinea, Fiji and seven different islands in Polynesia, as well as a population from Borneo [23]. This study also introduced a novel approach to correct for the ascertainment bias: the SNP discovery and depth of discovery were modeled by comparing summary statistics calculated on SNPs included on the Affymetrix 6.0 genotyping array to summary statistics calculated for the ENCODE sequence data from populations originally used in the SNP ascertainment scheme. This information was then incorporated as a prior into a Bayesian framework to test competing demographic models and infer demographic parameters. This study not only quantified the admixture in Polynesians as about 85% Asian and 15% New Guinean ancestry (with Borneo shown to be a better proxy for a parental population than Han Chinese, which were used to estimate admixture proportions in previous studies) but also showed that after initial settlement, Fijians received additional gene flow from Near Oceania, which did not spread further into Polynesia (see Figure 4), as also suggested by some archeological findings [91]. It was estimated that Fijians have about 63% Polynesian and 37% New Guinean ancestry. An ABC simulation-based approach was used to infer times of admixture, and for Polynesians, the admixture was estimated at approximately 3,000 years ago, while for Fijians at approximately 500 years ago. Both dates are in rough agreement with the dates inferred using a wavelet transform analysis-based approach on the same data [36] and are supported by archeological evidence [91]. The time estimate for Fiji unequivocally suggests additional gene flow from New Guinea well after the initial occupation of Remote Oceania.

**Figure 5:**
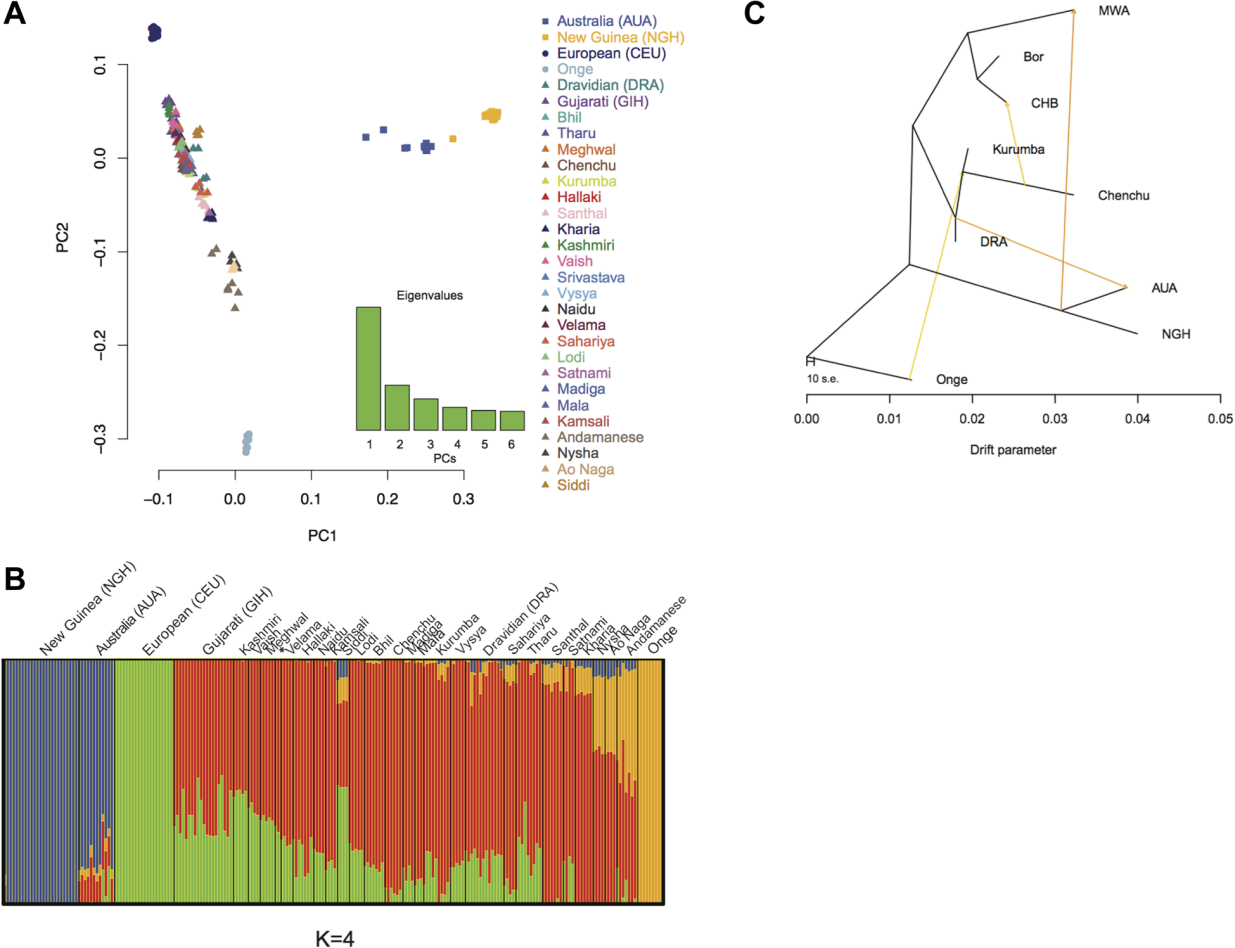
**Results of the PCA, ADMIXTURE, and TreeMix analyses, indicating gene flow from India to Australia. (A)** PCA of aboriginal Australians (AUA), highlanders of Papua New Guinea (NGH), Europeans (CEU), and 26 Indian populations. PC1 is driven by differences between the populations of Sahul and Eurasia. PC2 reflects a north-to-south gradient of European ancestry observed in Indian groups, with the southernmost group being the Onge, a Negrito population from the Andaman islands. **(B)** Population structure estimated using ADMIXTURE for *K* = 4. Each vertical bar represents an individual, and each color describes the proportion of each individual’s genome that comes from one of the four hypothetical ancestral populations (*K*). **(C)** Population graph obtained with TreeMix. First, the maximum likelihood tree of the nine populations included in the analysis was inferred, and then, migration events were added to the tree sequentially, until a graph with the smallest residuals was found. The graph that best fits the data has four inferred migration edges. Populations included are: AUA, NGH, Onge, Mamanwa (a Negrito group from the Philippines; MWA), East Asians (Chinese, CHB), Island Southeast Asians (Borneo, BOR), Indian populations: Chenchu, Kurumba, and Dravidian speakers from South India (DRA). Reprinted with permission from Pugach *et al*. [68].

Quite remarkably, despite the scope of their expansion, the Austronesians have left no genetic traces in mainland Australia. In fact, before genome-wide data became available, it was widely believed that following the initial colonization event, aboriginal Australians remained completely isolated from the rest of the world, until the arrival of the Europeans late in the eighteenth century. Studies of mtDNA [59,92] and NRY [93] variation have suggested a possible connection with India in the Holocene, but it was not until genome-wide data for the aboriginal Australians became available that this connection was substantiated further [68]. The study was based on around 1 million SNPs genotyped in aboriginal Australian samples from the Northern Territories, highlanders of Papua New Guinea, 26 populations from India and 11 populations from Island Southeast Asia (ISEA), as well as the HapMap populations. The gene flow from India to Australia was demonstrated via four independent analyses (PCA, ADMIXTURE, f4 statistic, and TreeMix) (Figure 5), and the date of this admixture was estimated to be approximately 4,200 years ago, that is, well before European contact. Since some pre-European trade probably existed between the northeastern coast of Australia and Indonesia [94], the scenario of indirect gene flow via ISEA was also considered, but no signal of Indian ancestry in populations of ISEA was discovered. Interestingly, the estimated date of admixture coincides with the time of the introduction of dingo [95], the first appearance of microliths (small stone tools) [96], and other changes documented in the Australian archeological record. It is therefore possible that these changes in Australia were associated with the migration from India, although this remains a controversial issue [97-99].

Since the sample of aboriginal Australians analyzed in this study came from the northwestern part of the continent, it would be interesting to investigate to what extent the Indian connection is shared throughout the Australian continent. The only other genome-wide study of aboriginal Australians was based on samples from the southeastern part of Australia (the Riverine area of western New South Wales) [67] and failed to discern any signal from India, but this is most likely because the study did not include any populations from India and hence had no adequate comparative data. On the other hand, the analysis of the Australian genome sequence did find indications of genetic relationships with groups from India, but the presented conclusion was that this signal represents some genetic ancestry in the Australian genome sequence that could not be assigned to any existing population [71].

In addition to the aforementioned insights into the history of past migrations that have shaped the history of Oceania, genome-wide data were useful in revealing finer population structure in Polynesia and in the highlanders of Papua New Guinea [23]. Unlike general patterns of population structure, which tell a story of ancient demographic events, such fine-scale structure is often indicative of existing social practices, like marrying within a group that shares the same language. For example, the sampled individuals from New Guinea, although they came from two neighboring villages, were clearly separated according to their language group (Huli *vs*. Angal-Kewa, both from the Engan branch of the Trans-New Guinea languages) both in the PCA and in the STRUCTURE-like clustering algorithm Frappe. Fine structure was also evident in Polynesia, as PCA of just the Polynesian samples revealed a separation between the Cook Islanders and the others along the first principal axis, while PC2 roughly differentiated non-Cook-Island samples according to their island of origin. In this case, the presence of fine-scale structure is probably best explained by geography and inter-island isolation.

### The impact of Austronesian expansion on Island Southeast Asia

By the time of the out-of-Taiwan migration, Island Southeast Asia had already been populated for tens of thousands of years. The first anatomically modern humans came to this region as part of the ‘southern-route’ out-of-Africa migration. Genetic evidence based on mtDNA, NRY, and autosomal markers suggests that there were additional dispersals into ISEA, possibly from mainland Asia, before the arrival of the Austronesians [100-103]. Austronesian languages are thought to have arisen in Taiwan [75], and today, they are widespread and spoken in the Philippines, Indonesia, Southeast Asia, and Madagascar (as well as in Polynesia and coastal New Guinea). To what extent was this dramatic spread of languages and a transition to agriculture the result of a large-scale expansion of people, or was it merely a cultural diffusion? Were the indigenous pre-Neolithic foraging populations of ISEA simply replaced or assimilated? Two recent genome-wide studies that analyzed data from the International Human Genome Organization (HUGO) Pan-Asian SNP Consortium and additional Austronesian- and Papuan-speaking populations from across Indonesia, Philippines, mainland Southeast Asia, and Papua New Guinea [104,105] have greatly contributed to our understanding of the genetic impact of the Austronesian expansion on populations of ISEA.

Geographically, western Indonesia (which includes the main islands of Borneo, Sumatra, and Java and surrounding smaller islands) lies on the Sunda Shelf, which was exposed during the last ice age (up to approximately 8,000 years ago), linking the islands of western Indonesia to the Asian continent. Eastern Indonesia is separated from the western Indonesia by a deep water channel known as Wallace’s Line which runs between the islands of Borneo and Sulawesi. Island Sulawesi and two archipelagos, Nusa Tenggara and the Moluccas, lie between the Sunda and Sahul (joint New Guinea-Australia landmass) shelves.

**Figure 6:**
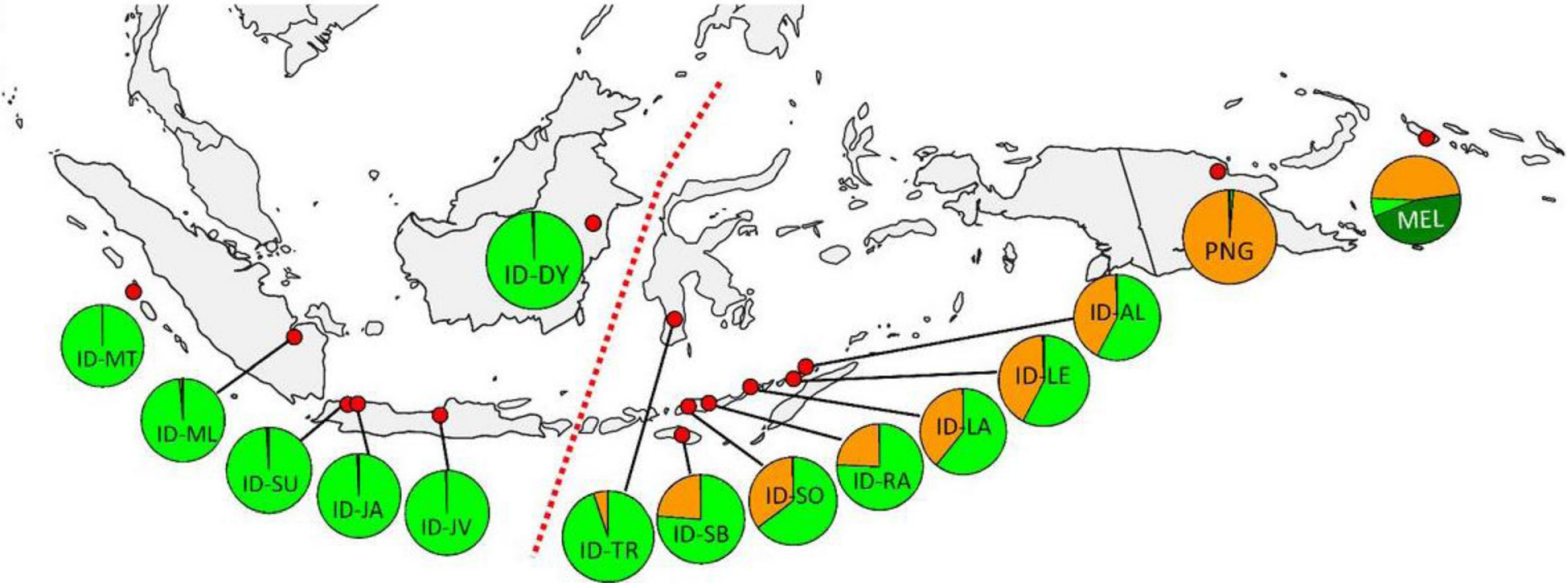
**Geographical distribution of Asian and Papuan genetic components across Indonesia.** Red dots on the map are sampling locations. Each circle graph represents a population sample, with the frequency of the genetic components inferred by STRUCTURE analysis (ID, Indonesian; MT, Mentawai; ML, Malay; SU, Sundanese; JA, Javanese; JV, Javanese; DY, Dayak; TR, Toraja; SB, Kambera; RA, Manggarai; SO, Manggarai; LA, Lamaholot; LE, Lembata; AL, Alorese; PNG, Papuan and MEL, Melanesian). Red dashed line denotes Wallace’s biogeographic line. Reprinted with permission from Xu *et al*. [104].

It has been shown previously based on mtDNA and NRY data [102,106] that east Indonesian populations are of dual Papuan and Asian descent. Yet, it was only when genome-wide data became available that it became possible to analyze the pattern of distribution of Asian ancestry and estimate the date of this historical mixing, thereby resolving the debate on pre-Austronesian *vs*. Austronesian origins of the Asian ancestry in Indonesia. The pattern that has emerged from the analysis is that the Papuan ancestry gradually increased (while the Asian ancestry decreased) from west to east across Indonesia (Figure 6), with the lowest proportion (5.1%) of Papuan ancestry being observed in the Toraja population of south Sulawesi (the closest population to the Wallace’s line in the dataset), while the Alorese - the population closest to New Guinea, exhibited the highest proportion (55.4%) of Papuan ancestry [104]. This same pattern was observed with a different dataset with samples from the Nusa Tenggaras and the Moluccas. The time of admixture was estimated separately in these two datasets and via two independent methods. The results obtained with the two datasets were very consistent with each other and suggested admixture first happened in the western part of eastern Indonesia approximately 5,000 years ago and only later (approximately 3,000 years ago) in the islands closer to New Guinea. These results are in excellent agreement with linguistic and archeological evidence for the time of the arrival of Austronesian languages and material culture in Indonesia [55,74-77] and refute the idea that the Asian ancestry observed in eastern Indonesia is unrelated to and predates Austronesian expansion and that the spread of Austronesian languages could be explained by cultural diffusion alone. Furthermore, the cline both in proportions of Austronesian ancestry and the dates of admixture strongly suggest that the spread of Austronesian-speaking farmers across Indonesia happened in the eastward direction.

**Figure 7:**
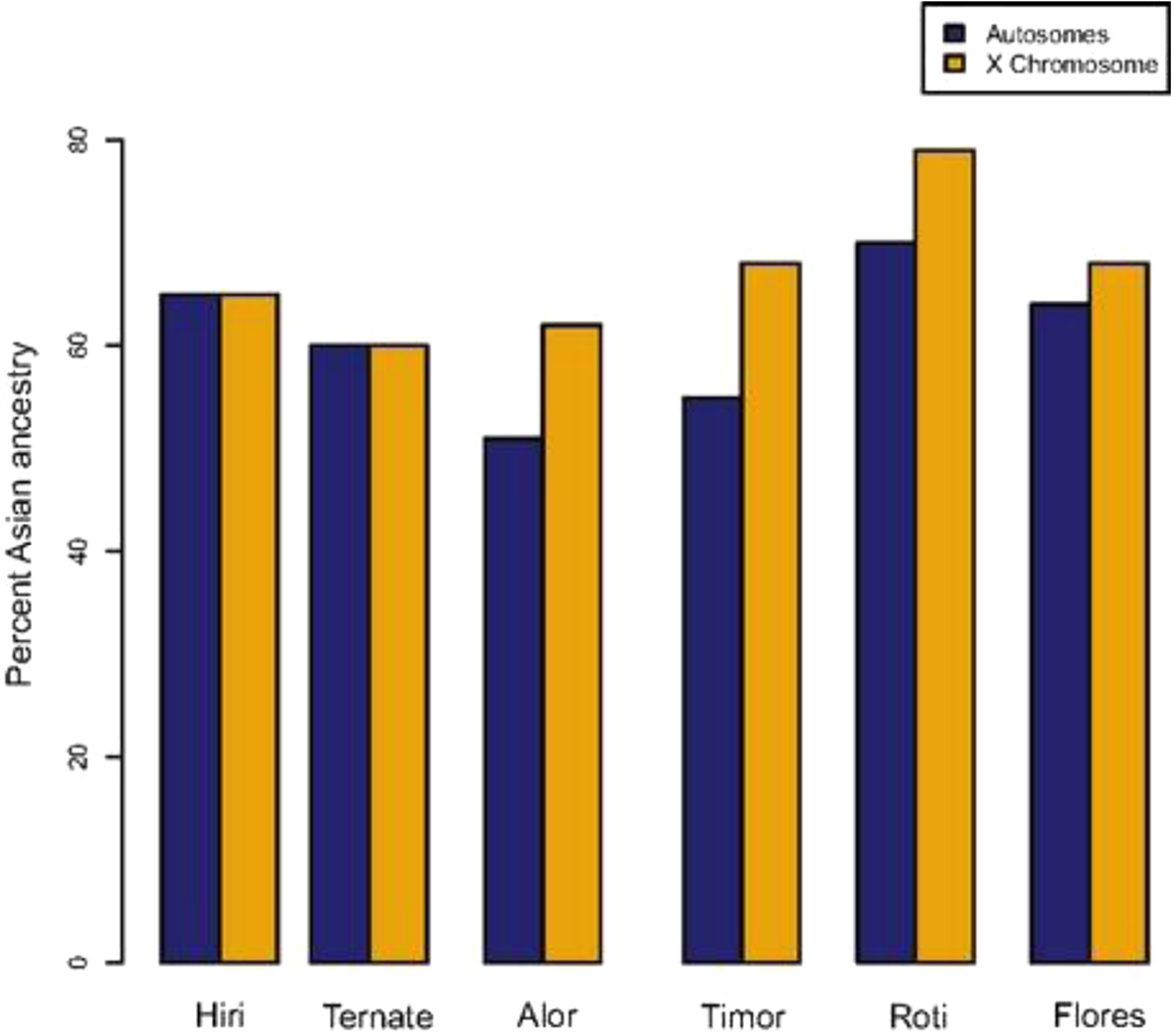
**Admixture estimates for autosomes** ***vs*** **. X chromosome in the Nusa Tenggara (Alor, Timor, Flores, Roti) and the Moluccas (Hiri, Ternate) populations of Indonesia.** Reprinted with permission from Xu *et al*. [104].

Another valuable insight came from the analysis of the admixture rates on the autosomes and the X chromosome in the Nusa Tenggara and the Moluccas populations. The samples from the Nusa Tenggaras, which came from Austronesian-speaking groups, showed a higher frequency of Asian ancestry on the X chromosome relative to the genome-wide estimates, suggesting that the admixture in these groups was sex-biased, with a greater contribution from Asian women. This pattern however is not seen in the Moluccas, where the sampled groups were Papuan speakers (Figure 7). These sex-related differences in the admixture between Papuan and Austronesian groups are again consistent with the hypothesis that the Austronesian groups were matrilocal [86,87], as also addressed in the previous section.

**Figure 8:**
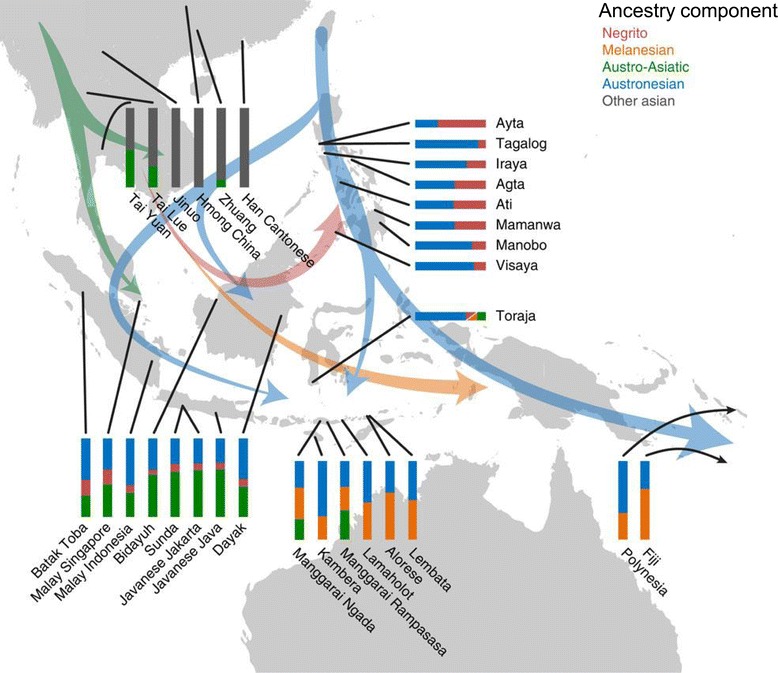
**Locations and best**-**fit mixture proportions for Austronesian**-**speaking and other populations, with suggested possible directions of human migrations.** For Toraja, it was not possible to distinguish between Negrito and Papuan (Melanesian) ancestry and this component is shown as red/orange. Reprinted with permission from Lipson *et al*. [105].

The importance of large-scale human migrations in the prehistory of ISEA was further illustrated by a study which demonstrated that ISEA has seen a succession of human migrations as populations of ISEA trace their ancestry to multiple sources [105]. The study also introduces a new method to analyze SNP chip data. This novel method, *MixMapper 2.0*, is relatively unaffected by ascertainment bias [107] and uses allele frequency correlations to construct an unadmixed phylogenetic tree and then sequentially adds to this tree admixed populations, inferring from the data the best placement, admixture proportions, and sources of ancestry for each admixed population in the dataset (contribution from multiple sources of ancestry is allowed). When applied to a dataset of 31 Austronesian-speaking and 25 other groups from the HUGO Pan-Asian SNP Consortium and the CEPH-Human Genome Diversity Panel (HGDP), the method identified four ancestral components differentially distributed in populations of ISEA (Figure 8). The Austronesian component, which is most closely related to Taiwan aboriginals, is ubiquitous and is observed in all populations of ISEA (and Polynesia). The Papuan (Melanesian) component is restricted to East Indonesia and Polynesia (as shown before, see above). The Negrito component is present in variable proportions in all populations in the Philippines and is also observed, albeit at lower frequencies, in all populations of western Indonesia, whereas it is completely absent in eastern Indonesia. The Austro-Asiatic component occurs among Austro-Asiatic speakers on mainland SEA and intriguingly is also prevalent in western Indonesia, but not seen anywhere else in ISEA, except in the Manggarai people of island Flores in eastern Indonesia (close to the Wallace’s Line)

This study also estimated dates of admixture in ISEA using the software *ALDER* [35], which uses a linkage disequilibrium statistic to estimate times of admixture. However, the dates obtained are substantially more recent than those estimated for the arrival of Austronesians in ISEA based on archeological and linguistic evidence [74-77], and more importantly, these dates are substantially more recent than the dates inferred via two different methods (one of which is also based on LD) using the same data for eastern Indonesia, Polynesia, and Fiji [23,104]. Although the authors of this study suggested that the more recent dates of admixture reflect more recent gene flow that is not detected by other methods, it is also possible that there is some inherent limitation or bias to the method; further studies are needed.

Because the dates of admixture are inconclusive, it is difficult to infer the sequence of events that led to such a substantial Austro-Asiatic ancestry in western Indonesia. The authors offer three explanations. The first scenario implies that Austronesian expansion proceeded via mainland SEA, where this genetic component was picked up and subsequently brought to western Indonesia. However, this scenario does not explain the complete absence of the Austro-Asiatic signal in eastern Indonesia. Also, if the Austro-Asiatic component arrived in western Indonesia concomitantly with the Austronesian component, then we would expect the proportions of these two components in the descendent populations to be correlated; this remains to be shown. Another explanation involves recent admixture from mainland SEA, which cannot be ruled out at this point. The third possibility is that at the time of Austronesian migration, the Austro-Asiatic ancestry was already widespread in western Indonesia, which in our opinion is the most likely scenario, as the islands of western Indonesia, but not eastern Indonesia, were up until around 8,000 years ago connected to mainland SEA (forming Sundaland), and thus, the Austro-Asiatic ancestry observed in western Indonesia could be related to the indigenous population of Sundaland. Further studies of correlations in ancestry, and dating of admixture signals, should shed light on the origins of the Austro-Asiatic ancestry in western Indonesia. For additional reading on the population history of the region, we provide the reader with the references to other interesting and relevant studies [108-110].

### The colonization of the New World

North and South America were the last continental regions to be colonized by humans. Current evidence suggests that humans first entered the New World via the Bering land bridge about 15,000 years ago [111], but questions remain as to how many migrations there might have been and how much genetic ancestry each separate migration contributed to contemporary Native American populations. The linguistic picture is controversial; there is general agreement on two language families: Na-Dene (also known as Athabascan), spoken across northwestern North America and by some groups in the American Southwest (such as Apache and Navajo) that migrated there in recent times, and Eskimo-Aleut, spoken by native groups distributed from eastern Siberia, across the Aleutian Islands and Arctic North America, and into Greenland. It is all of the remaining 600 or so languages that are controversial, as some linguists lump these all into a single family called ‘Amerind,’ whereas other linguists see evidence for as many as 30 (or even more) distinct, unrelated language families, along with dozens of language isolates.

**Figure 9:**
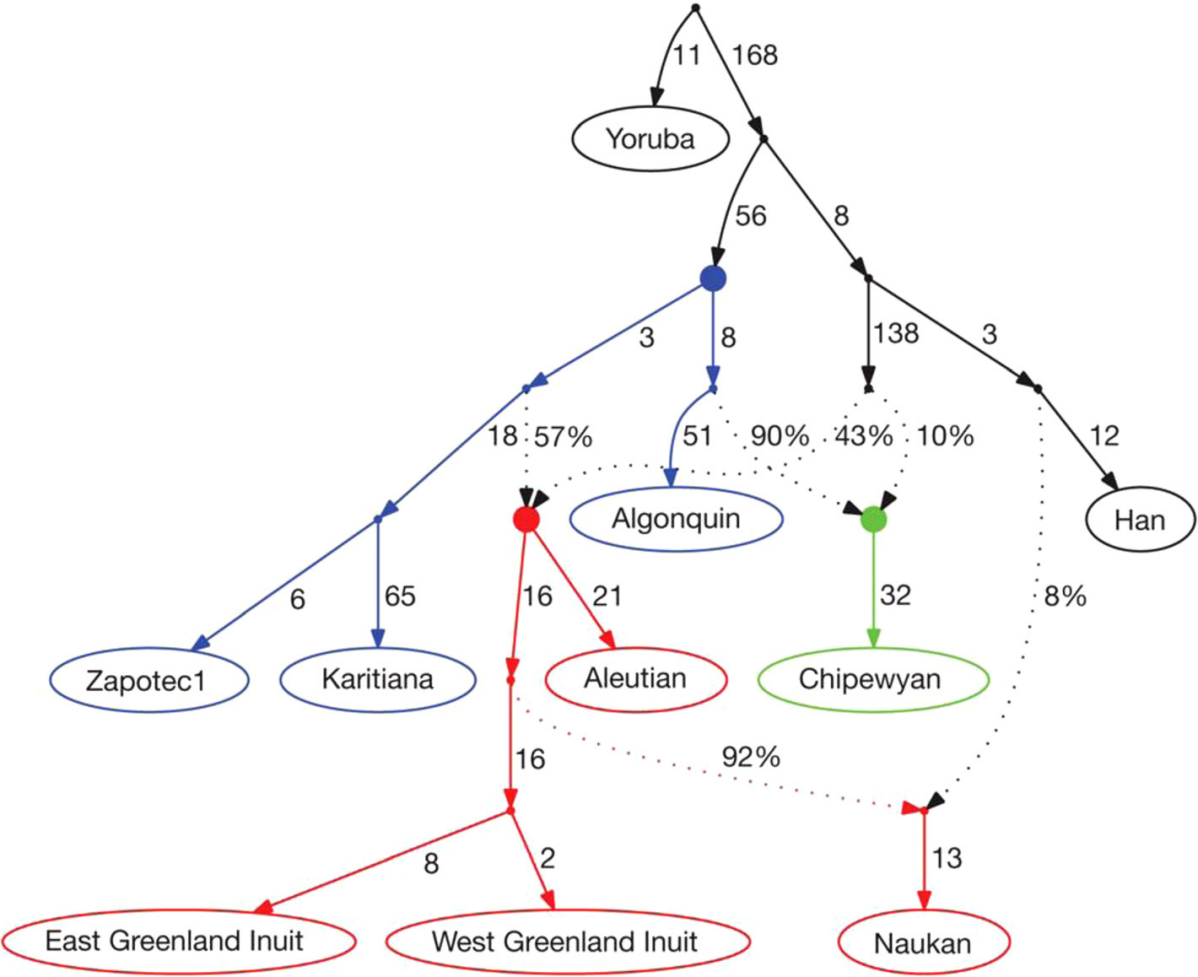
**Migration and admixture history of Native American populations, inferred from genome**-**wide SNP data.** Solid points indicate inferred ancestral populations, solid lines indicate descent with numbers indicating genetic drift (in units proportional to genetic distance), and dotted lines indicate admixture events with associated percentage of ancestry contributed. Red indicates Eskimo-Aleut groups; green indicates a Na-Dene group; and blue indicates Amerind groups. Reprinted with permission from Reich *et al*. [112].

Most of the genetic evidence that has been used to investigate the colonization of the New World comes from either mtDNA and Y chromosome studies or from ancient DNA and hence is discussed elsewhere in this issue. However, there is some relevant genome-wide data from contemporary Native American groups. A study of genome-wide SNP data from 52 Native American populations [112] found evidence for (at least) three gene flow events from Asia to the New World (Figure 9): one associated with Na-Dene groups; one associated with Eskimo-Aleut groups; and one associated with all other groups in the analysis (which, for convenience, we will refer to as Amerind, without implying any associated linguistic uniformity of such groups). Briefly, the analysis involves fitting an admixture graph (which depicts both a branching history of populations as well as migration events) to the data, using various statistics, to arrive at the best-fitting model of population history. Note that while this is the best-fitting model and none of the statistics indicated a poor fit of the model to the data, it is nonetheless not possible to test if the best-fitting model is significantly better than other models, because the statistics used to fit the admixture graph to the data are all highly correlated. Note also that with this approach, there is no information about the time of inferred population divergence or migration events or about population size changes; other approaches (such as ABC simulations) would be needed for such additional inferences. Nonetheless, the admixture graph presents some interesting results. The ‘Amerind’ ancestry diverged first, while the Na-Dene and Eskimo-Aleut ancestry stems from a common ancestral Siberian source population. The Eskimo-Aleut groups have nearly equal amounts of Amerind and Eskimo-Aleut ancestry, while the single Na-Dene group in the analysis has nearly 90% Amerind ancestry and only 10% ancestry shared with the Eskimo-Aleut ancestor (Figure 9). The analysis also identified a back migration from North America to Siberia involving the ancestors of the Naukan Yupik, who subsequently admixed with Chukchi populations.

While the results of this study are consistent with previous genetic evidence suggesting three major migrations to the New World, there are some important caveats. The sampling of North American populations was limited to just one Na-Dene group and three Amerind groups, so it remains to be seen if the admixture graph depicted in Figure 9 can account for all of the ancestry in contemporary Native American populations. A recent study of genome-wide SNP data in indigenous Mexican populations found that the genetic differentiation between some groups was as large as that observed between European and Asian populations [113]. Whether all of this genetic differentiation within Mexico can be explained by a single migration and subsequent isolation and drift, or whether it instead reflects the legacy of multiple migrations, is an interesting question for further study.

**Figure 10:**
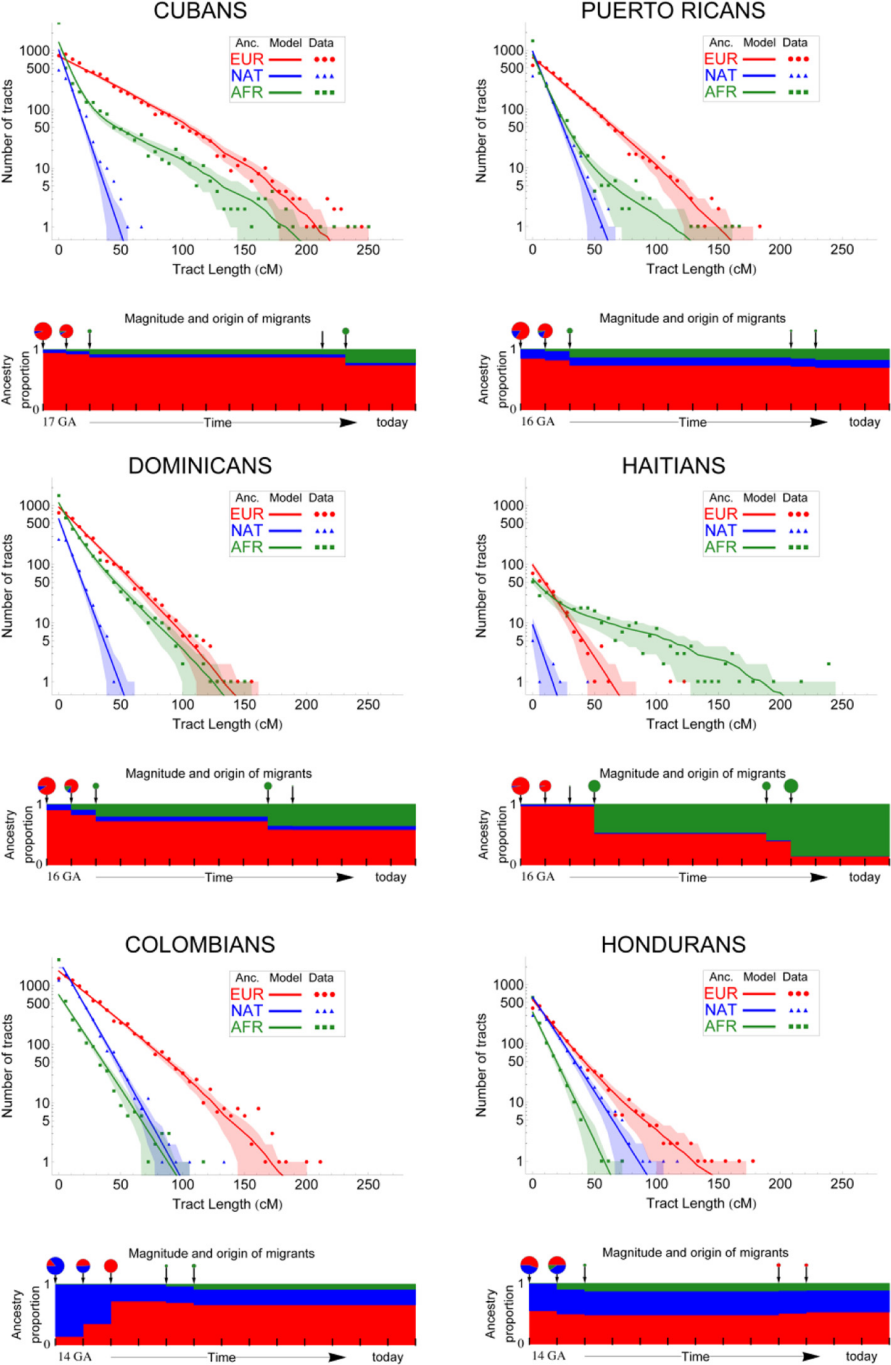
**Admixture from European and African sources in the demographic history of native Caribbean populations.** Shown are the relative proportions of Native American (blue), European (green), and African (red) ancestry, based on lengths of inferred ancestry tracts. For each population, below the ancestry tract plots are shown the admixture events and associated times and ancestry contribution. Reprinted from Moreno-Estrada *et al*. [114].

There are other questions of interest concerning Native American populations that are being addressed with genome-wide data. For example, since the arrival of Europeans and with the introduction of the African slave trade, European and/or African ancestry can be detected in many Native American populations. Over what time periods was such ancestry contributed, what were the source populations, and how much of an impact does this recent European and/or African ancestry have on Native American populations? Two studies have recently addressed these questions, one analyzing genome-wide SNP data in Caribbean populations [114], and the other analyzing genomic sequence data from three Native American populations in the 1000 Genomes Project [115]. Both studies analyzed the distribution of the number and length of chromosomal segments of different ancestries (ancestry tracts) to come up with the best-fitting model of admixture history (for example, Figure 10) and to identify potential source populations for the European/African ancestry. Interestingly, in the Caribbean, the European ancestry deviates markedly from contemporary Iberian ancestry (the presumed historical source of the European ancestry in the Caribbean), suggesting pronounced founder events during European colonization of the New World. Moreover, some populations exhibit two distinct pulses of African ancestry, coinciding with historical data for the onset and maximum impact of the African slave trade and with different sources in west Africa [113]. Thus, genome-wide data can contribute additional insights into historically attested admixture events.

### Genetic structure of Europe

The origins of modern Europeans remain contentious; for decades, anthropologists have tried to answer the question to what extent the Paleolithic hunter-gather populations known in Europe since around 45,000 years ago were replaced, assimilated, or have adopted the way of life of farmers, as agricultural practices and/or farmers started spreading across Europe from the southeast ca. 8,500 years ago. The most informative insights into the history of Europe have come from recent ancient DNA work [116-119], which shows that European history is far more complicated than previously anticipated and that all modern Europeans trace their origins to three, and not two, sources of ancestry [118]. These consist of the Paleolithic and Neolithic ancestries mentioned above, as well as a third source of ancestry that appears to have originated from north Eurasia occurring subsequent to the advent of agriculture [118]. Since this chapter focuses on insights from modern populations rather than from ancient DNA, we provide the ancient DNA references for the interested reader and instead briefly mention the evidence that comes from the genome-wide genetic studies of modern-day populations. It should be kept in mind that the early events that have shaped the history of Europe have largely been obscured by the extensive migrations which happened more recently.

Two comprehensive studies of genome-wide variation that densely sampled across a geographic continuum of Europe [120,121] revealed that although the autosomal gene pool of Europe overall has very little structure, it shows a striking correlation with geography. Both studies used principal component analysis to summarize genetic variation, and the two-dimensional representation of the result revealed that the genetic map of Europe almost completely coincides with the geographic map. Both studies report a genetic continuum between Europeans, with populations closer to each other geographically appearing closer to each other genetically. This pattern is expected under the ‘isolation-by-distance’ models, where the genetic similarity in a two-dimensional space decays with distance if there is small-scale local gene exchange between neighboring populations [122]. Nevertheless, sampling a large number of loci in combination with dense geographic sampling affords an unprecedented resolution on a local scale. In particular, Novembre *et al*. [121] were able to show that individuals in Switzerland despite being located on a genetic continuum could be somewhat separated based on the language they speak, with the Italian-, French-, or German-speaking people showing closer relationships within a Swiss sample according to the language spoken in that part of the country. Furthermore, based on the genetic data alone, over 90% of individuals could have been successfully placed within 700 km of their place of origin, and over 50% of people within 310 km [121]. However, it should be kept in mind that these results are based on a rather ‘artificial’ subsample of Europeans, namely those that have all four grandparents coming from the same locale (village, town, or city), and hence are not representative of all Europeans.

This geographic structure of recent relatedness was further explored by a subsequent study which used the same dataset to infer genomic segments inherited from a recent common ancestor identical by descent (IBD). The study applied a new methodology based on the estimated lengths of these IBD blocks to relate these lengths to the ages of the most recent common ancestors [123]. As before, it was observed that mostly, it was the geographic proximity which determined the amount of IBD sharing, with the most IBD blocks shared by individuals belonging to the same population (albeit with a few exceptions explained by asymmetric gene flow from a smaller population into a larger one). As expected, as the geographic distance between the tested populations increased, a smooth decay of relatedness was observed. Nonetheless, even geographically distant European populations were shown to share ubiquitous common ancestry, and this ancestry was dated to within the past 1,000 years, leading to the conclusion that all Europeans are genealogically related over very short time periods. However, regional variation was also observed, notably the populations of the Italian and Iberian peninsulas appeared to share little recent common ancestry with the other European populations, and what little is shared was dated back to 2,500 years ago. This pattern is explained by the authors as either stemming from the old substructure apparently present in Italians, which was not erased by recent migrations or from the existence of certain geographic barriers (for example, the Pyrenees) which limited the gene flow to and from the Iberian peninsula [123]. Furthermore, a slight decrease in the mean heterozygosity and increase in linkage disequilibrium in the south-to-north direction across Europe was also described [120].

In conclusion, the studies of genetic variation in Europeans show little overall genetic differentiation between populations, which could be the result of the homogenizing effect of recent migrations across Europe, yet reveal startling correspondence between genes and geography, even on a regional scale [124-127]. Given that the data for these three studies were generated on Affymetrix GeneChip 500 K array and hence are a subject to ascertainment bias, which mainly affects alleles present in populations at low frequency and hence are likely to stem from mutation events with a very localized place of origin, it is reasonable to expect that data collected in a more unbiased way (for example, whole genome sequences) will afford even greater resolution than that revealed by these studies.

## Conclusions

In this review, we have focused on a few of what we find to be the most interesting stories concerning human population history that have been illuminated by studies of genome-wide SNP data. One of the main messages is that while ascertainment bias is always an important concern with such data, there are ways to account for ascertainment bias in demographic analyses (or even take advantage of such bias, as for example, with the different ascertainment panels in the Human Origins Array). Another main message is that as we get better and better at detecting and dating admixture signals in genome-wide data [128], we find more and more evidence of admixture between different human populations (as well as between modern and archaic humans). This has important consequences for how we think about ourselves: the commonly held view that after initial dispersals, human populations settled down and were largely isolated until the time of European colonization is no longer tenable. Instead, the history of human populations has always involved migrations, dispersals, contact, and admixture, and we look forward to the stories that future genome-wide studies reveal about ourselves.

## Abbreviations

ABC: approximate Bayesian computation. A likelihood-free, simulation-based approach to statistical inference, used for estimation of demographic parameters and model selection
CEPH-HGDP: Human Genome Diversity Cell Line Panel
HUGO: International Human Genome Organization
IE: Indo-European languages
ISEA: Island South East Asia
LD: linkage disequilibrium. Non-random association of alleles among the polymorphic loci
mtDNA: mitochondrial DNA. A circular piece of non-recombining DNA of approximately 16,000 bp that is inherited exclusively from the mother
PC: principal components. In PC analysis, the first principal component captures as much of the variability in the data as possible, and each succeeding component accounts for the next highest variance possible, while being constrained to be uncorrelated with the preceding components
PCA: principal component analysis. A statistical method that is used to simplify a complex dataset by orthogonal transformation of correlated variables into a smaller set of uncorrelated variables known as principal components
SNP: single nucleotide polymorphism. A common variation in a DNA sequence that occurs when a single nucleotide in a genome is altered
STR: short-tandem repeat. A variable number of tandem repeated short sequence motifs
